# Fine spatial-scale variation in scavenger activity influences avian mortality assessments on a boreal island

**DOI:** 10.1371/journal.pone.0233427

**Published:** 2020-05-21

**Authors:** Megan J. Clarke, Erin E. Fraser, Ian G. Warkentin

**Affiliations:** Environmental Science Program, Memorial University of Newfoundland—Grenfell Campus, Corner Brook, Newfoundland and Labrador, Canada; CNRS - Universite de Pau et des Pays de l'Adour - E2S UPPA, FRANCE

## Abstract

Bird-window collisions are the second leading cause of human-related avian mortality for songbirds in Canada. Our ability to accurately estimate the number of fatalities caused by window collisions is affected by several biases, including the removal of carcasses by scavengers prior to those carcasses being detected during surveys. We investigated the role of scavenger behavior in modifying perceived carcass removal rate while describing habitat-specific differences for the scavengers present in a relatively scavenger-depauperate island ecosystem. We used motion activated cameras to monitor the fate of hatchling chicken carcasses placed at building (under both windows and windowless walls) and forest (open and closed canopy) sites in western Newfoundland, Canada. We recorded the identity of scavengers, timing of events, and frequency of repeat scavenging at sites. Using 2 treatments, we also assessed how scavenging varied with 2 levels of carcass availability (daily versus every third day). Scavenger activities differed substantially between forest and building sites. Only common ravens (*Corvus corax*) removed carcasses at building sites, with 25 of 26 removals occurring under windows. Burying beetles (*Nicrophorus* spp.) dominated scavenging at forest sites (14 of 18 removals), completely removing carcasses from sight in under 24 hours. Availability had no effect on removal rate. These findings suggest that ravens look for carcasses near building windows, where bird-window collision fatalities create predictable food sources, but that this learning preceded the study. Such behavior resulted in highly heterogeneous scavenging rates at fine spatial scales indicating the need for careful consideration of carcass and camera placement when monitoring scavenger activity. Our observations of burying beetle activity indicate that future studies investigating bird collision mortality near forested habitats and with infrequent surveys, should consider local invertebrate community composition during survey design. The high incidence of invertebrate scavenging may compensate for the reduced vertebrate scavenger community of insular Newfoundland.

## Introduction

Human modified environments can present unfamiliar hazards to local wildlife populations, having potentially detrimental impacts on the species involved [1,2,3]. Among the numerous causes of avian mortality directly linked to human activities (e.g. predation by cats, collisions with various structures, electrocution from power lines), collisions with windows are the second highest source with estimates ranging from 16–42 million mortalities annually in Canada [4] and 365–988 million in the US [5]. For the many species of conservation concern regularly documented as casualties in bird-window collisions, accurately quantifying the extent of losses attributable to this cause is important for the development of conservation actions [6].

Our ability to collect accurate data on human-related mortality within wildlife populations is constrained by several biases in the survey techniques used. One of the most important biases affecting mortality estimates is the removal of carcasses by scavengers or decomposers before a carcass survey is conducted [7]. Given that the scavenging of carcasses can occur via removal by vertebrate scavengers or through decomposition by invertebrate scavengers and microbes [8], there may be high spatial and temporal variability in scavenging rates depending upon differences in the abundance of the species involved, the extent of competition for carcasses among these species, and seasonal variation in activity levels [9,10]. Island settings, often characterized by restricted communities and a lower diversity of vertebrate scavengers than are found in mainland settings, create further variability [11]. Other scavenger species (e.g., invertebrates) can compensate for this functional loss of vertebrate scavenger species on islands, altering the relative contributions of vertebrate and invertebrate community members to scavenging, and consequently affecting the overall scavenging rate [11]. There are currently are no inclusive estimators available to account for carcass scavenging and other biases when examining human-related mortality events [12].

Evidence suggests that buildings with high window collision rates also have high scavenging rates, indicating a possible scavenger learning component in response to these predictable sources of food [7,12]. Flint and colleagues [13] found that scavengers focused their foraging activity where carcasses were deployed during scavenging trials; they hypothesized that the scavengers had become habituated to regular food availability along these transects [13]. Scavenging rates are thus likely also influenced by the ability of many scavenger species to learn and adapt their behavior in response to predictable sources of food [14]. However, we know of no studies that sought to explicitly investigate such habituation in the context of carcasses made available in association with bird-window collisions.

Our objective was to investigate scavenger activity in response to varying bird carcass availability across a range of site types, including at the base of buildings under windows (mimicking carcasses resulting from bird-window collisions), at the base of buildings along walls with no windows, and in nearby forested sites with both open and closed canopy cover. We hypothesised that the scavenging species present would vary among site types [9] and that scavengers would respond to the rate of carcass availability [12,13,14]. Specifically, we predicted that the scavenging species present would differ between the 2 broad site types, building and forest, and that consecutive scavenging events at a particular site by the same scavenger (as an indication of scavenger habituation) would be more likely to occur at sites with daily carcass deposition compared to infrequent deposition (i.e., every third day). Finally, we predicted that scavengers would remove carcasses sooner at building window sites when compared to wall sites, based on a previously learned response (i.e., habituation) to the predictable availability of carcasses associated with bird window collisions [13].

## Methods

### Study site

**Fig 1:**
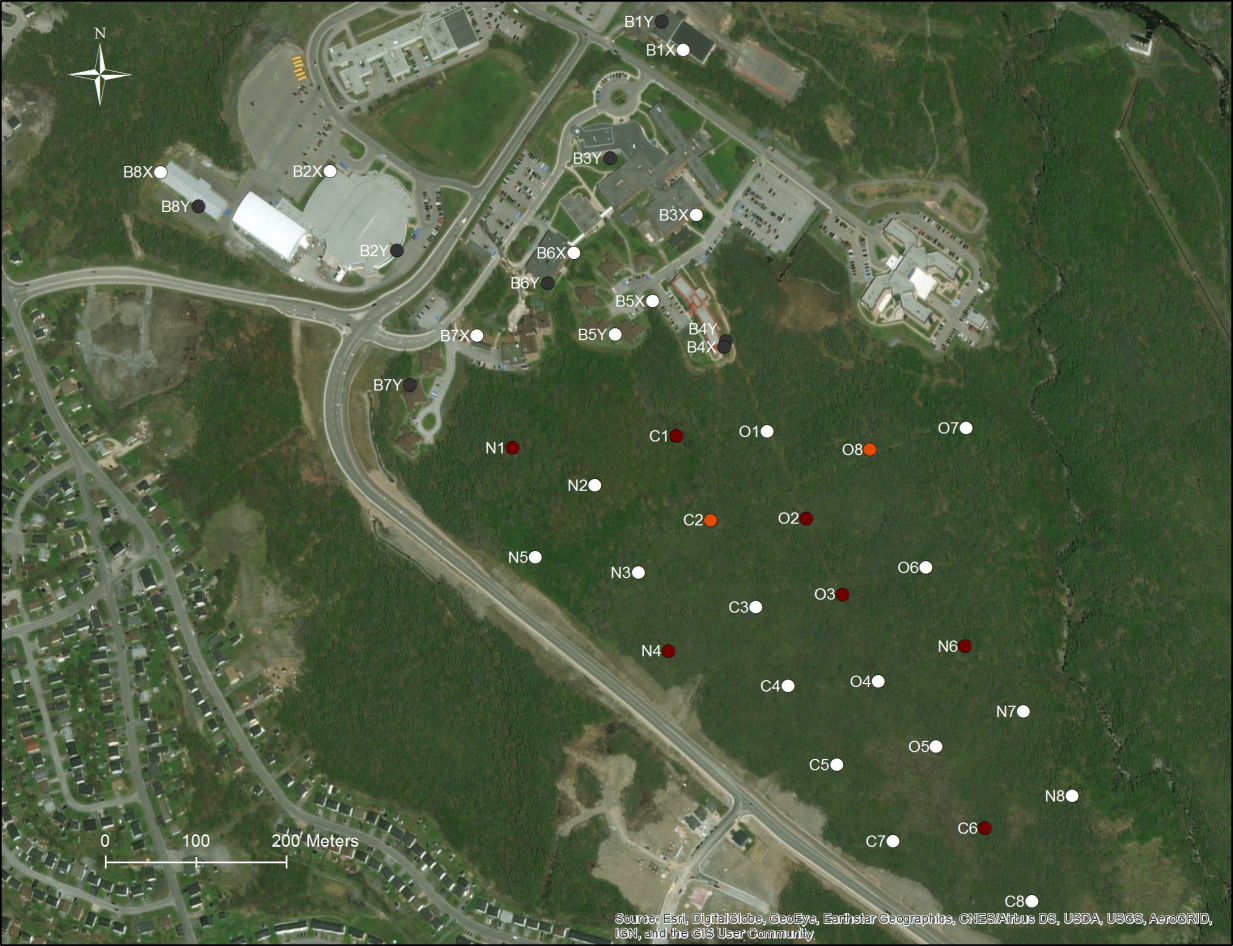
Survey sites used on and around Grenfell Campus, Memorial University of Newfoundland, Corner Brook, NL, Canada (48° 56’ 23.05” N; 57° 56’ 11.01” W). Grey markers indicate sites where a raven was the first scavenger detected, red markers indicate sites where a burying beetle was the first scavenger detected, orange markers indicate sites where a fox was the first scavenger detected, and white markers indicate sites where there were no scavenging events. Native carcasses: N = forest interior sites. Chicken carcasses: C = forest interior sites; O = forest trail sites; BY = building window sites; BX = building wall sites. Image generated using ArcGIS Online World Imagery basemap (Sources: Esri, DigitalGlobe, Earthstar Geographics, CNES/Airbus DS, GeoEye, USDA FSA, USGS, Aerogrid, IGN, IGP, and the GIS User Community).

**Fig 2:**
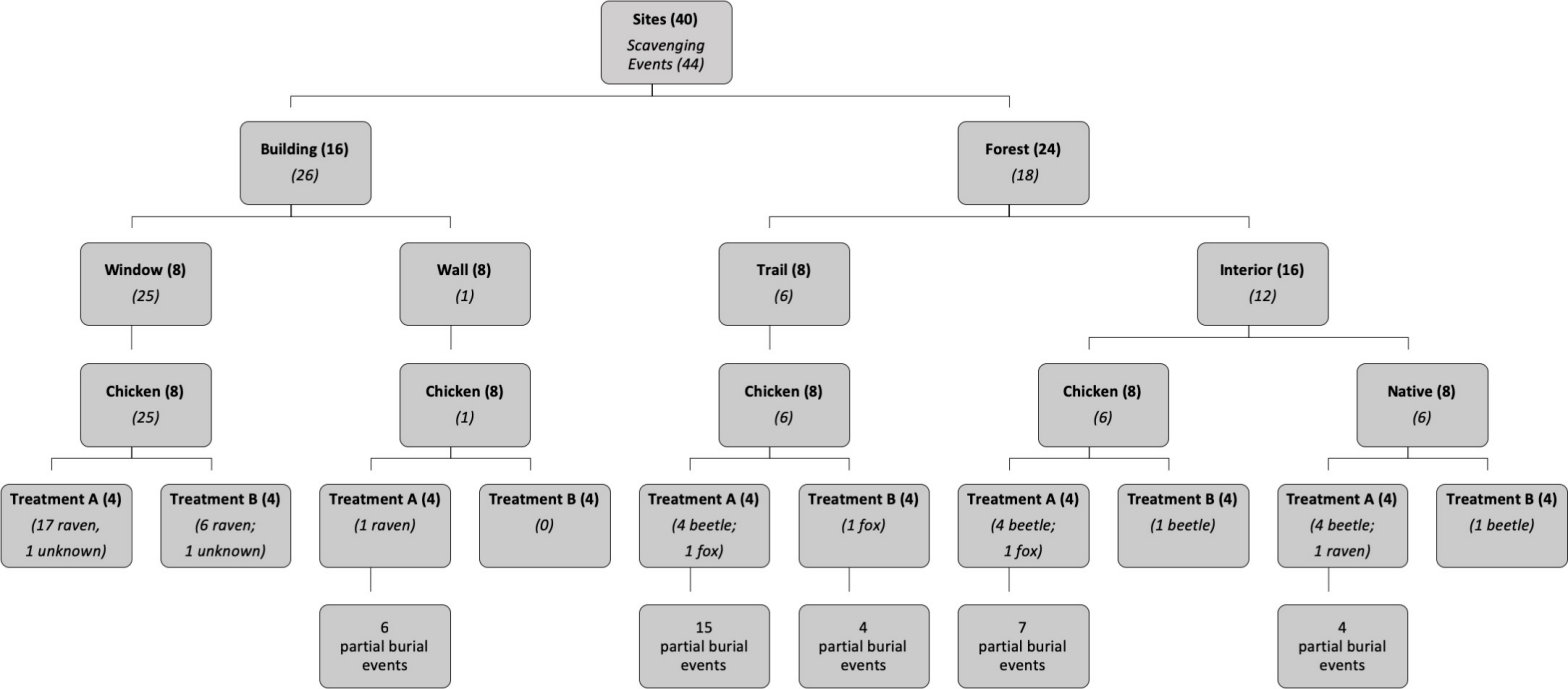
Schematic representation of study design and summary of scavenging results. Sampling occurred at 2 general site types (building versus forest), 2 specific site types within each of these (window versus wall and trail versus interior, respectively), 2 carcass types (hatchling chicken versus native species), and 2 treatments (A = carcass available daily; B = carcass available only every third day). We present number of sites (bold) and scavenging events (italics) for each combination. In addition to the reported full scavenging events, we also report the number of partial burial events by burying beetles for each combination in the bottom row of boxes; the latter values were not included in the number of full scavenging events for boxes above this level.

We conducted surveys of scavenging activity at 40 sites within a total sampling area of 0.55 km^2^ on the Grenfell Campus of Memorial University of Newfoundland and in the adjacent conifer-dominated boreal forest of the Western Newfoundland Ecoregion [15] in Corner Brook, Newfoundland, Canada (48° 56’ 23.05” N; 57° 56’ 11.01” W; 86 m elevation) (Fig 1). Grenfell Campus includes primarily 2–3 story high buildings surrounded by low-density urban areas and has a documented low frequency of bird-window collision mortality [16]. We selected 8 buildings on and around Grenfell Campus for surveys and identified 2 survey sites at each building (Fig 2): an area along an external wall of the building which had windows (window area: 24.5 ± 28.4 m^2^ (mean ± SD); wall width: 22.3 ± 16.6 m; wall height: 9.20 ± 2.69 m) and an area along a wall without any windows (at least 6.3 m in width at the base, wall width: 28.3 ± 19.6 m; wall height: 7.90 ± 4.00 m). Additionally, we identified 24 sites in the forested area adjacent to Grenfell Campus where: (i) we assessed scavenging activity in 2 forest site types, and (ii) compared scavenging rates for carcasses of naturally occurring songbirds versus those of hatchling domestic chickens (Fig 2). Eight of these forest sites were located on a footpath and had limited forest canopy cover and high visibility to avian scavengers and the other 16 sites were located in the forest interior and had greater cover and more limited visibility to avian scavengers. Each of the 24 forest sites and the 8 buildings were at least 100 m away from one another and all forested sites were at least 100 m away from any buildings.

### Field methods

We used motion activated cameras (6 Moultrie M-1100i 12MP Digital Game Cameras and 4 Bushnell Trophy Cam HD Essential E2 12MP Trail Cameras) to monitor the fate of bird carcasses placed at each of the 40 sites. Both camera types were programmed to take a burst of 3 photos when triggered by movement in the detection area of the sensor, with a minimum 5 second delay between triggers. We conducted 4, 15-day trials starting on 29 May, 15 June, 2 July, and 6 August 2018, respectively. To assess whether carcass availability affected scavenging, we used 2 treatments. For treatment A, carcasses were available daily and were replaced within 24 hours of a scavenging event, or when the carcass became noticeably degraded. For treatment B, we placed carcasses at sites every third day and removed those remaining approximately 24 hours after each placement. We randomly assigned 4 sites from each of the site types (building window, building wall, forest trail, forest interior chicken, forest interior native) to treatment A and the remaining sites were assigned to treatment B, resulting in a total of 20 sites for each treatment (Fig 2). All treatments and site types were represented evenly in each of the 4 trials.

On day 1 of each 15-day trial, we placed motion activated cameras at every site, either attached 0.65 ± 0.14 m above the ground to a metal stake (building sites) or a tree (forest sites) and oriented facing north to prevent direct sunlight from affecting the camera’s infrared trigger [17]. On day 2 of each trial, we placed a bird carcass (either chicken hatchling or native bird) at each site, 2.2 ± 0.7 m away from the camera and 1.1 ± 0.2 m away from the base of the wall at building sites. All carcasses were secured loosely to the ground with a wooden golf tee pushed through the wing to slow their removal by scavengers, thus increasing the likelihood that the scavenging events would be captured by the cameras. We visited sites daily, starting between 10:00–11:00. During subsequent daily site visits, any scavenging events were noted, memory cards were replaced for all cameras and carcasses were removed as required by the treatment or replaced if needed as a result of a scavenging event. We defined a scavenging event as having occurred when a carcass was removed completely from the site (vertebrate scavenging event), or the carcass was completely buried underground (invertebrate scavenging event). A partial burial event was defined as a carcass still visible at the site of placement after a burying attempt by an invertebrate scavenger. Bird-window collision surveys [16] were conducted daily at each building used in a given trial, to quantify existing bird-window collision mortalities.

We later reviewed all photos, recorded the timing of any animal or scavenging activity and identified any active scavenging species. If scavenging events occurred by the same species of vertebrate scavenger at the same site on consecutive carcass placement days (5 consecutive events for treatment A, 3 consecutive events for treatment B), we stopped the experiment at that site to avoid habituation by scavengers to artificial food sources. Invertebrates found scavenging carcasses in the field were collected, promptly frozen, and later identified to species [18].

Carcasses of hatchling chickens that had died of natural causes were donated by a local hatchery (Country Ribbon Farms, St. John’s, NL); these were stored in a freezer until use. Hatchlings weighed between 22.34 g and 59.30 g (39.10 ± 8.48 g). These naturally bright yellow carcasses were dyed with edible food colouring (Sweet Sugarbells Edible Coloring Sprits–Black; Wilton Icing Colour–Black and Brown) before placement in the field so their color was more representative of the native songbird population. The native bird carcasses were collected from Grenfell campus over the past 5 years as bird-window collision mortalities and had been stored in a freezer. Native birds weighed between 5.79 g and 25.10 g (13.08 ± 5.33 g) (list of species used in S1 Table). Carcasses were removed from the freezer and thawed overnight before placement in the field the following day. All methods were conducted according to Newfoundland and Labrador Scientific Research Permit WLR2018-11, Environment Canada—Scientific Permit SS2159, and Animal Care Protocol 18-01-EF. The permits were received from The Government of Newfoundland and Labrador—Department of Fisheries and Land Resources and the protocol was approved by the Memorial University Institutional Animal Care Committee.

### Statistical analysis

To assess the factors affecting time to first scavenging event for chicken carcasses (data from limited native species carcasses not included), we used a two-way ANCOVA. The response variable was the proportion of time until first scavenging event out of the total potential carcass availability per trial (312 hours for treatment A and 120 hours for treatment B), with treatment type (A or B) and experiment site type (building window, building wall, forest trail, forest interior) as predictors and trial (represented by first ordinal day of the trial, where day 1 = 1 January) as a covariate. We arcsine square root transformed the proportion data to minimize outliers and adjust for a right skew; the site X treatment interaction was not statistically significant and visual inspection of residuals indicated homogeneity of variance, supporting the use of an ANCOVA. Unlike vertebrate scavenging events, the exact time of full burial by invertebrate scavengers was not captured by the cameras. Due to variability in the literature regarding the daily activity patterns of the invertebrate species collected [18], we arbitrarily defined the time of total burial as the time when the burial was identified in the field during the daily site visit. We used a non-parametric Mann-Whitney U test to compare the time until first scavenging event for native versus chicken hatchling carcasses, with carcass type as the categorical variable. All analyses were conducted using R version 3.5.2 with statistical significance noted at α = 0.05.

## Results

### Site specific patterns

From a total of 360 opportunities for scavenging to occur (measured as days of carcass availability at 40 sites over the 4 trials), we detected such events 44 times at 18 sites. An equal number of native and chicken carcasses (6 each) were scavenged from forest interior locations, and there was no difference in the proportion of time until first scavenging event between carcass types (Mann-Whitney U test: *n* = 16, *W* = 39, *p* = 0.43; hours of carcass availability until a scavenging event or the end of the trial, for native carcasses (mean ± SD) 150.0 ± 95.8 hrs (range 24–312 hrs); for chicken carcasses 175.5 ± 86.8 hrs (range 120–312 hrs). During the study period a single natural bird-window collision mortality was observed on the last day of the final trial in August, under a window expanse at building B4 (Fig 1).

The scavengers detected at building and forested sites differed. Ravens were the only animals identified scavenging at building sites (24 of 24 removals; for 2 scavenging events the individual could not be identified) (Fig 2). Burying beetles dominated scavenging at forested sites (14 of 18 removals), along with 3 detections of fox scavenging and 1 raven (Fig 2); note that 6 of these removals were of native species carcasses (5 by beetles and 1 by a raven) and that these events were not included in the ANCOVA reported below. In addition to the full burial events by burying beetles, we also detected 36 partial burial events (Fig 2). We identified 4 species of burying beetles: *Nicrophorus sayi*, *N*. *vespilloides*, *N*. *defodiens*, and *N*. *investigator*. Additional animal species detected in photos included: moose (*Alces alces*), American robins (*Turdus migratorius*), red squirrels (*Tamiasciurus hudsonicus*), eastern chipmunks (*Tamias striatus*), a feral cat (*Felis catus*), and snowshoe hare (*Lepus americanus*). There were no indications that any of these species scavenged the bird carcasses.

### Time until first scavenging event

There was a difference in the proportion of time until first scavenging event (chicken hatchling carcasses only) among the 4 different site types (Two-way ANCOVA; *F*_*(3*,*23)*_ = 4.51, *p* = 0.01), but not between treatments (*F*_*(1*,*23)*_ = 3.12, *p* = 0.09). Because there was no effect of trial as a covariate (*F*_*(1*,*23)*_ = 0.39, *p* = 0.54), we re-ran the analysis using a two-way ANOVA with no covariate to enable the use of a post-hoc Tukey HSD test. The proportion of time until first scavenging event at building window sites was significantly shorter than that at both building wall and forest interior sites (*p* = 0.019 and *p* = 0.022 respectively), but not different from the forest trail sites (*p* = 0.053) (hours of carcass availability until a scavenging event or the end of the trial, for window sites (mean ± SD) 75.8 ± 91.5 hrs (range: 0.6–271 hrs); for all other site types 174 ± 96.1 hrs (range 1.2–312 hrs).

### Consecutive scavenging events

We identified consecutive vertebrate scavenging events at only 5 sites (scavenging events by the same species occurring on consecutive carcass placement days); all these sites were at building windows with raven scavengers. Of the 5 sites, 3 were from treatment A (2, 3, and 6 consecutive scavenging events respectively), and 2 were from treatment B (both with 3 consecutive scavenging events). We stopped the trial early at 2 of these building window sites (one from both treatment A and B), after reaching the limit of consecutive scavenging events by the same species (ravens) as dictated by our animal care protocol. There were also consecutive invertebrate scavenging events at 3 of the forested sites with chicken hatchling carcasses, and these were restricted to trials 2 (2 sites; 4 and 2 consecutive events respectively) and 3 (1 site; 2 consecutive events), and all were from treatment A. Since burying beetles were collected for identification, the repeat scavenging events at these sites were caused by different individual scavengers.

## Discussion

We observed consistent scavenging of bird carcasses within our island study area but there were marked site-related differences for the scavengers involved: ravens were responsible for all on-campus scavenging events around buildings, while burying beetles dominated forest scavenging events. Carcass placement location on campus was important, with ravens showing a clear tendency to scavenge carcasses placed beneath windows rather than at walls with no windows, and this pattern held for all consecutive on-campus scavenging events as well. Likewise, these scavenging events under windows occurred more quickly than those at other site types but there was no clear effect of carcass availability on scavenging rate, regardless of site. We found no differences in scavenging activities between carcass types at forest interior sites. There is no *a priori* reason to predict a wall versus window X carcass type interaction influencing scavenging activity around buildings, although future studies should confirm that this is indeed born out by testing.

Avian scavengers are well suited to visually searching for foraging opportunities in the open habitat around building sites, while mammals have an advantage over avian scavengers in locating carcasses via olfactory cues in the understory of the forest [19]. Our observations of vertebrate scavenging events support this general pattern, with all but one avian (raven) scavenging event occurring in the relatively open campus environment, and the few mammalian (fox) scavenging events occurring only in the forest. Based on the early summer presence of a pair of ravens and the mid-summer appearance of raven fledglings on the Grenfell Campus, we suspect that there was a raven nest located in the forest edge adjacent to one of the study sites (B7Y; Fig 1). Ravens commonly nest in urban/suburban areas and defend relatively small nest territories of 300 m^2^-1.2 km^2^ in these human influenced landscapes [20,21], an area which could encompass all of the building sites used in the present study. While neither member of this breeding pair was marked, it is possible that these birds were responsible for many or all of the raven scavenging events detected. This demonstrates the potential for a particularly fine scale of heterogeneity in scavenging rate across the landscape, with the presence of 1 or 2 individual scavengers potentially dominating scavenging activities in their vicinity. It was surprising that none of the other animals detected with our cameras scavenged the bird carcasses available, especially the feral cat which was detected frequently at both building and forest sites. Feral cats are commonly documented opportunistic scavengers in urban/suburban environments [9,12,14], however the cat photographed in the present study was frequently looking right at the camera in the photos, behavior that has previously been interpreted as indicating that the presence of the cameras may deter cats from removing carcasses [17].

The literature associated with mortalities and scavenging events resulting from bird-window and other types of collisions rarely make mention of invertebrate scavengers [7,9,13]. Consequently, we made no *a priori* predictions about their involvement in this experiment and unexpectedly found that burying beetles were the dominant scavengers (9 of 12 events using hatchling chicken carcasses) at the forested sites and also were engaged in 36 partial burial events across all site types. Many islands, including Newfoundland, have a reduced vertebrate scavenging population with several prevalent mainland scavengers being absent (in North America: raccoon (*Procyon lotor*), Virginia opossum (*Didelphis virginiana*), etc.) [14]. Under such circumstances, it has been suggested that invertebrate scavengers can functionally compensate for reduced vertebrate populations on islands and maintain the essential ecosystem service of carcass removal [11]. We observed burying beetles fully burying both chicken hatchling and native carcasses in less than 24 hours; a relevant time frame for bird-window collision surveys, especially if buildings are located near or within the types of forested sites where we found full burial events occurring in this study [16].

Burying beetles occurred most often at sites with deep, loose soil which aids in the success of reproductive activities that are associated with carcass burial. Our observation of no full burial events and only 6 partial burial events around buildings on campus may have resulted from the soil being packed by foot traffic in this developed area, and thus a more difficult substrate for burying carcasses [22]. The absence of partial burials under building windows may have been because ravens scavenged carcasses at these locations so quickly that the beetles were not able to locate and bury the carcasses. The presence of many partially buried carcasses in the present study suggests that invertebrate scavenging could be even more relevant for less frequent (multiple days to a week) mortality count surveys and especially those in forested settings, as are common along powerline corridors and at wind turbine farms [17,23].

We observed consecutive beetle scavenging events only during the middle 2 of the 4 trials (time period: 15 June through 17 July), even after individual beetles were removed from the sites on consecutive days, suggesting a high level of temporal variability in beetle activity. Burying beetles have a short reproductive period during early- to mid-summer and are strongly attracted to food sources (rotting carcasses) during this time [18]. The seasonal variability in our findings highlights the importance of considering local invertebrate life cycles when accounting for scavenging rates in animal mortality counts. These results suggest that diligent searching is needed for animal mortality surveys in and around forested sites to ensure that buried or partially buried carcasses resulting from invertebrate scavenging are taken into consideration.

Corvids are capable of complex learning [24] and many examples exist of members of this group quickly developing effective food gathering strategies in novel situations. Observations of common ravens show that they learn to associate with other predators such as wolves, foxes, and seabirds, and will scavenge the spatially and temporally unpredictable kills and caches of these other species [25,26,27]. The strong tendency for on-campus scavenging events by ravens to occur under windows (23 of 24 events) suggests a pre-existing awareness by these scavengers of windows as sites with food availability, and potentially a rapidly learned response to the subsequent presence of carcasses (i.e., consecutive scavenging events occurring at these locations). Conversely, the absence of initial scavenging and repeated carcass removal events at building sites without windows suggests that the ravens exhibited a lack of food discovery in these novel locations over the time frame of the present study. Additionally, there was no evidence of habituation by foxes, the only other vertebrate observed scavenging. Foxes are commonly documented opportunistic scavengers that likely have the ability to learn about regularly available food [28]. Our findings may reflect the low density of foxes in the area, as well as the higher cost and lower likelihood of locating carcasses by olfactory cues for mammalian scavengers in the dense forested habitats where they were detected [29].

## Conclusions

Estimates of mortality rates for birds encountering the hazards associated with human modified environments are clearly affected by scavenging. Our findings support the conclusions of previous studies that habitat characteristics may correspond with scavenging activity and influence the composition of the scavenging community. Likewise, the presence of scavengers with *a priori* knowledge of predictable food sources may lead to highly heterogeneous scavenging rates and rapid removal of carcasses even from sites with typically low mortality rates. The implications of our findings for future study of mortality rates where scavenging of carcasses is possible include: (1) the need for careful cataloging of the scavenger community density and distribution in the study area to understand the potential mechanisms for losses to scavengers, (2) the value of understanding the broader and finer spatial scale movement patterns of these scavengers to account for habitat and space use differences among scavengers, (3) the need to consider carcass placement on a fine spatial scale relating to potentially habituated scavengers, and (4) the importance of determining the timeframe for scavenger removal of carcasses in the context of survey frequency. We also suggest incorporating motion activated cameras into regular protocols when monitoring scavenging activity, as these aid in identifying the exact timing of scavenging events and the species involved. Lastly, invertebrates in this study fully and partially buried small carcasses in less than 24 hours and were particularly active at forested sites. Carcass search protocols would benefit from assessing the activity/abundance of local invertebrate communities and including diligent search methods to account for carcass removal by invertebrate scavengers, when investigating human related avian mortality events. The activity of invertebrate scavengers may be especially important to consider for mortality estimates around anthropogenic developments that are embedded within a forest environment (e.g. power lines, wind energy facilities) and for those protocols with longer periods between mortality surveys.

## Supporting information

S1 Table List of avian species used as native bird carcasses in this experiment. (XLSX)

S1 Dataset (XLSX)
